# Effects of the e-Motivate4Change Program on Metabolic Syndrome in Young Adults Using Health Apps and Wearable Devices: Quasi-Experimental Study

**DOI:** 10.2196/17031

**Published:** 2020-07-30

**Authors:** Ji-Soo Lee, Min-Ah Kang, Soo-Kyoung Lee

**Affiliations:** 1 Keimyung University Daegu Republic of Korea; 2 Gyeongnam Center for Infectious Disease Control and Prevention Changwon Republic of Korea

**Keywords:** metabolic syndrome, telemedicine, mobile apps, preventive care, wearable electronic devices

## Abstract

**Background:**

The health behaviors of young adults lag behind those of other age groups, and active health management is needed to improve health behaviors and prevent chronic diseases. In addition, developing good lifestyle habits earlier in life could reduce the risk of metabolic syndrome (MetS) later on.

**Objective:**

The aim of this study is to investigate the effects of the e-Motivate4Change program, for which health apps and wearable devices were selected based on user needs. The program was developed for the prevention and management of MetS in young adults.

**Methods:**

This experimental study used a nonequivalent control group. In total, 59 students from 2 universities in Daegu, Korea participated in the study (experimental group n=30; control group n=29). Data were collected over 4 months, from June 1 to September 30, 2018. The experimental group received a 12-week e-Motivate4Change program intervention, and the control group received MetS education and booklets without the e-Motivate4Change program intervention.

**Results:**

After the program, the experimental group had significantly higher scores for health-related lifestyle (*t*=3.86; *P*<.001) and self-efficacy (*t*=6.00; *P*<.001) than did the control group. Concerning BMI, there were significant effects by group (*F*=1.01; *P*<.001) and for the group × time interaction (*F*=4.71; *P*=.034). Concerning cholesterol, there were significant main effects for group (*F*=4.32; *P*=.042) and time (*F*=9.73; *P*<.001).

**Conclusions:**

The e-Motivate4Change program effectively improved participants’ health-related lifestyle scores and self-efficacy, and significantly reduced their BMI and cholesterol levels. The program can be used to identify and prevent MetS among young adults.

## Introduction

### Background

Chronic disease is a significant factor of disability as well as a major cause of death for nearly 60% of the global population [1]. Metabolic syndrome (MetS) is a serious risk factor for heart disease, stroke, and type 2 diabetes. There are numerous indicators of MetS, including hypertriglyceridemia, high blood sugar, and abdominal obesity; the incidence rate of MetS is steadily increasing [2]. Concurrently, MetS significantly lowers individuals’ quality of life (QOL) [3-6], which is defined as one’s level of acceptance in relation to the ideals, interests, expectations, and norms of one’s culture and value system [7]. Health-related QOL, in particular, is a personal evaluation of the physical and psychological impact of a disorder [8]. Although patients use this indicator to demonstrate their satisfaction level with their own functionality, scholars use it to analyze the average health level and the impact of health care among a wide range of populations [6].

The World Health Organization (WHO) reported that the incidence rate of chronic disease is not only related to social and economic factors but also to individuals’ eating habits and physical activities such as drinking and smoking [9]. Sedentary lifestyle, high-calorie diet, sweetened drinks, low economic status, and saturated fats are key risk factors that increase the risk of obesity; however, regular physical activity is known to lower individuals’ risk [10,11]. People who are habitually active, less sedentary, and have better cardio-pulmonary functions are less obese and thus have lower incidence rates of MetS [12]. Further, one exemplary study reported that better knowledge of one’s own disease increases medical accessibility and has a positive impact on the treatment process and self-care, thus improving individuals’ lifestyles [13].

The incidence rate of MetS is notably high among children and adolescents in the United States [14]. Further, the morbidity rate is associated with the obesity rate among young adults, which is also highly related to numerous adult diseases including arteriosclerosis, cardiovascular disease, cancer, and diabetes. Thus, appropriate prevention and intervention is needed in all related fields [14]. One can significantly lower the risk of and prevent MetS by controlling diet habits, physical activity, smoking, drinking, and stress levels [10,15,16]. In particular, men aged 30 to 39 years and women aged 20 to 29 years have worse health activity compared to other age categories, and thus require more active and focused health management to improve their health habits and prevent chronic diseases [17]. Since most health habits become more solidified in mid to older age, it is important to develop a MetS prevention program and prepare the foundation for appropriate health habits among young adults.

Several studies have analyzed the relationship between the incidence rate of MetS and its risk factors such as smoking, drinking, physical activity, depression, and environmental and demographic factors [18]. Moreover, one study revealed that an unhealthy daily lifestyle increases the risk of cardiocerebrovascular diseases and that improved habits act as preventive measures [19]. Further, the Third Report of the National Cholesterol Education Program Expert Panel in Europe reported that it is important to identify patients with MetS and treat them to prevent cardiocerebrovascular diseases [20].

Previous studies on MetS intervention programs examined ubiquitous health (uHealth) nutrition education that focused on increasing fruit and vegetable intake [21] and community-based health education, both of which appeared effective [22]. The uHealth nutrition education initiative is an online self-monitoring program, and data are collected during each session, and are then analyzed cumulatively [21]. A previous cross-sectional study in rural Taiwan successfully reduced the mortality rate from MetS by applying an appropriate Health City plan [23]. The Health City plan for Miaoli County, Taiwan integrated public and civil departments, nongovernmental organizations, and community resources to develop a model of “must move” and “healthy diet” as part of a sustainable development strategy. Thus, an appropriate health promotion project may effectively reduce the threat of death by MetS [23]. However, most previous studies on MetS and its interventions focused on adults aged >30 years; consequently, more studies about young adults are needed.

With the rapid development of information and smart technology, the demand for mobile health (mHealth), which uses mobile technology such as health apps to prevent disease and improve health, has increased [24,25]. Currently, there are numerous apps that support users’ health management. However, users report an unsatisfactory experience when they download the desired app and do not find the content of the app useful or relevant, making it difficult to use the app in the long run [26]. According to a user experience survey, 25.3% of users delete an app once they stop using it [27].

Wearable devices are a key subfield of mHealth. Wearable devices can constantly and easily monitor one’s physical activity, and they can provide tailored feedback through synchronized apps [28]. According to previous international studies, wearable devices can improve the lifestyle of patients with chronic diseases [24,26,28]. Numerous companies including Fitbit, Xiaomi, and Samsung produce wearable devices, and the market for devices that primarily manage health is steadily increasing [29]. According to Wijsman and colleagues [30], health interventions using wearable devices increase the daily physical activities of older adults and improve their metabolism.

However, there are some problems with mHealth. First, there is a lack of entertaining aspects that can motivate sustained use. Second, the system that directly delivers the information and professional health care guidelines is incomplete [27]. Other reported problems include a lack of visual information, inconvenient data entry, and one-sided feedback [26]. Therefore, to motivate users and foster active participation, it is important to increase users’ self-efficacy, which provides significant intrinsic motivation [31].

Self-efficacy is closely related to how well a person can control and perform specific activities under certain situations. Based on previous studies, health-related self-efficacy is a key motivator for one to continue physical activity; [32] thus, it serves as an important determinant of healthy lifestyle promotion [33]. Previous studies also reported a significant correlation between young adults’ health activity and self-efficacy [34,35].

Therefore, this study aims to improve on existing intervention programs by actively using wearable devices and mobile apps and increasing entertaining factors and motivation. To support sustained health activity, we developed a tailored program based on the specific needs of young adults to increase their self-efficacy and general health-related QOL.

### Objectives

The specific goal of our study is to develop a program (e-Motivate4Change) using health apps and wearable devices selected based on user's needs, and analyze its impact on MetS prevention among young people. In addition, we sought to determine the program’s effect on participants’ health-related lifestyle, self-efficacy, and QOL.

## Methods

### Study Design

This experimental study used a nonequivalent control group and pre- and post-test design. The experimental group completed a presurvey, the e-Motivate4Change program intervention, and three postsurveys. The control group received a pamphlet on MetS and completed three postsurveys. Data were collected over 4 months, from June 1 to September 30, 2018.

**Figure 1:**
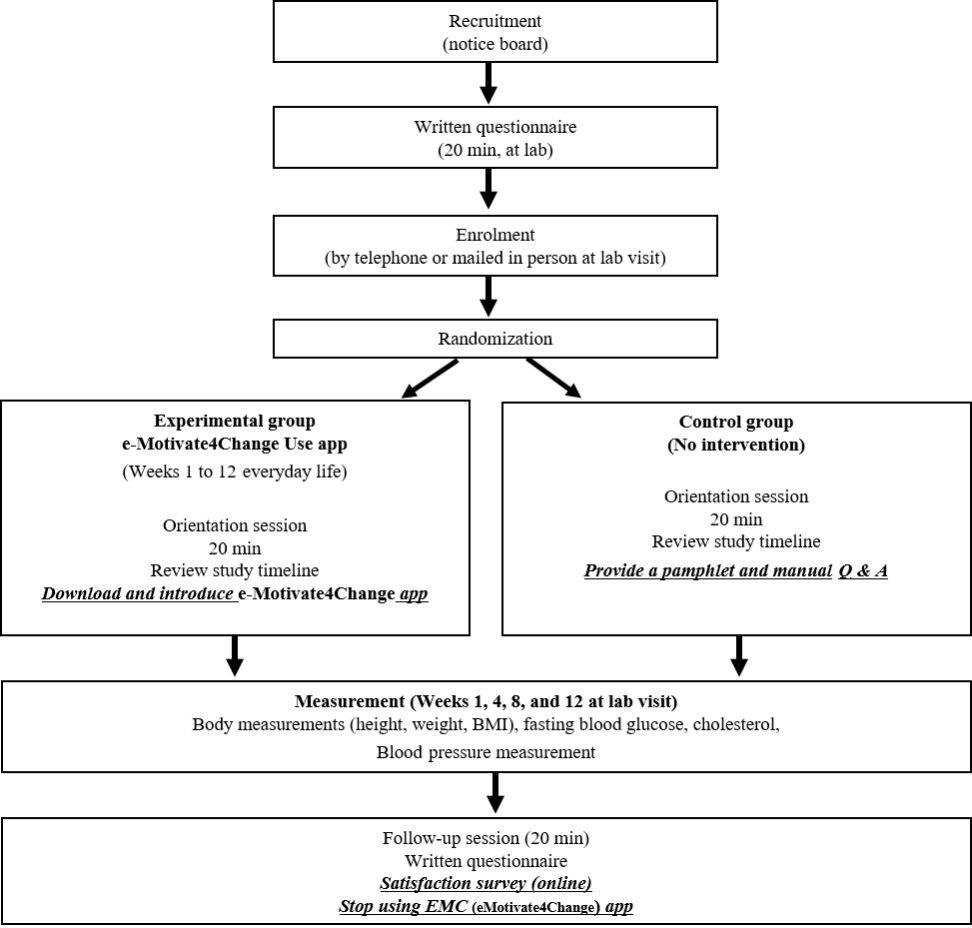
Flowchart of participants through each stage of the study.

The questionnaire consisted of general characteristics, healthy lifestyle promotion, self-efficacy, and QOL. To collect physiological indicators, we measured participants’ BMI, blood pressure, blood sugar, and cholesterol level. The general flow of the study is shown in Figure 1.

### Participants

Nursing students from Daegu University and Keimyung University who volunteered to participate and met the criteria were recruited. G*power (Version 3.1.7) was used to calculate the appropriate sample size with an effect size of 0.50, a significance level of .05, and a qualification level of 80%. The program indicated that at least 27 participants were required.

Anticipating a dropout rate of 20%, we recruited 60 participants (30 for each group) [36]. During the study period, one student from the control group opted out owing to a leave of absence; therefore, the data from 59 students were analyzed.

### Research Procedure

Participants were allocated to either the experimental or control group and the questionnaires were distributed. To measure healthy lifestyle promotion, we used Kang’s modified version [31] of Walker’s health-promoting lifestyle profile [37]. We used a self-efficacy measurement tool developed by Sherer and colleagues [38] to measure self-efficacy. We also used the translated version of the WHO’s QOL instrument (short version; WHOQOL-BREF) to measure QOL. It took approximately 10 minutes to complete the questionnaire.

**Figure 2:**
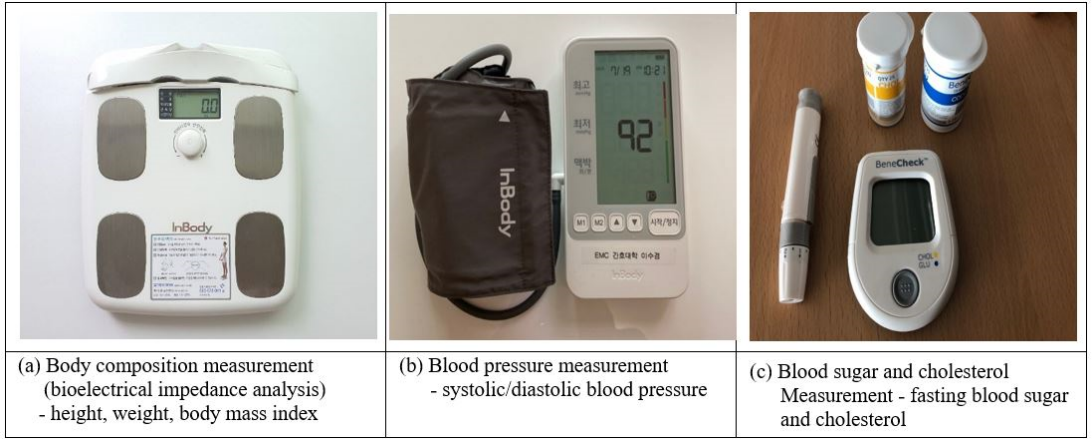
Physical and physiological assessment tools.

After completion, participants’ height, weight, body composition, blood pressure, blood sugar level, and cholesterol level were measured (Figure 2). BMI was measured using a body composition analyzer (InBody 3.0; Biospace), which uses bioelectrical impedance analysis. According to the categorization of Asian adults’ BMI, as reported by the Korean Society for the Study of Obesity, participants with a BMI from 18.5 to 24.9 kg/m^2^ are “normal,” while a BMI from 25.0 to 29.9 kg/m^2^ indicates “slightly overweight,” and a BMI over 30.0 kg/m^2^ indicates “obese” [39].

Participants were not provided with any written results of their body measurements since participants could freely check their status through the program. Additionally, the case report form, which is the electronic document of individuals’ information as stated in the research design, provided the necessary data to the team. All participants were thoroughly educated on how to complete the case report form.

The e-Motivate4Change program was implemented for 10 minutes with the experimental group to increase their knowledge about MetS and promote health-related activities. Participants could also ask questions. In addition, 5 minutes were allowed for questions and answers. The control group was not provided with the e-Motivate4Change program; they only received a pamphlet. After the e-Motivate4Change program, the experimental group were asked to complete the questionnaire on healthy lifestyle promotion, QOL, and self-efficacy. We then measured their BMI, blood sugar level, cholesterol, and blood pressure again. Participants also completed an online satisfaction survey using a separate site on the e-Motivate4Change app. The control group completed the same surveys and health measurements.

### Intervention Design

**Figure 3:**
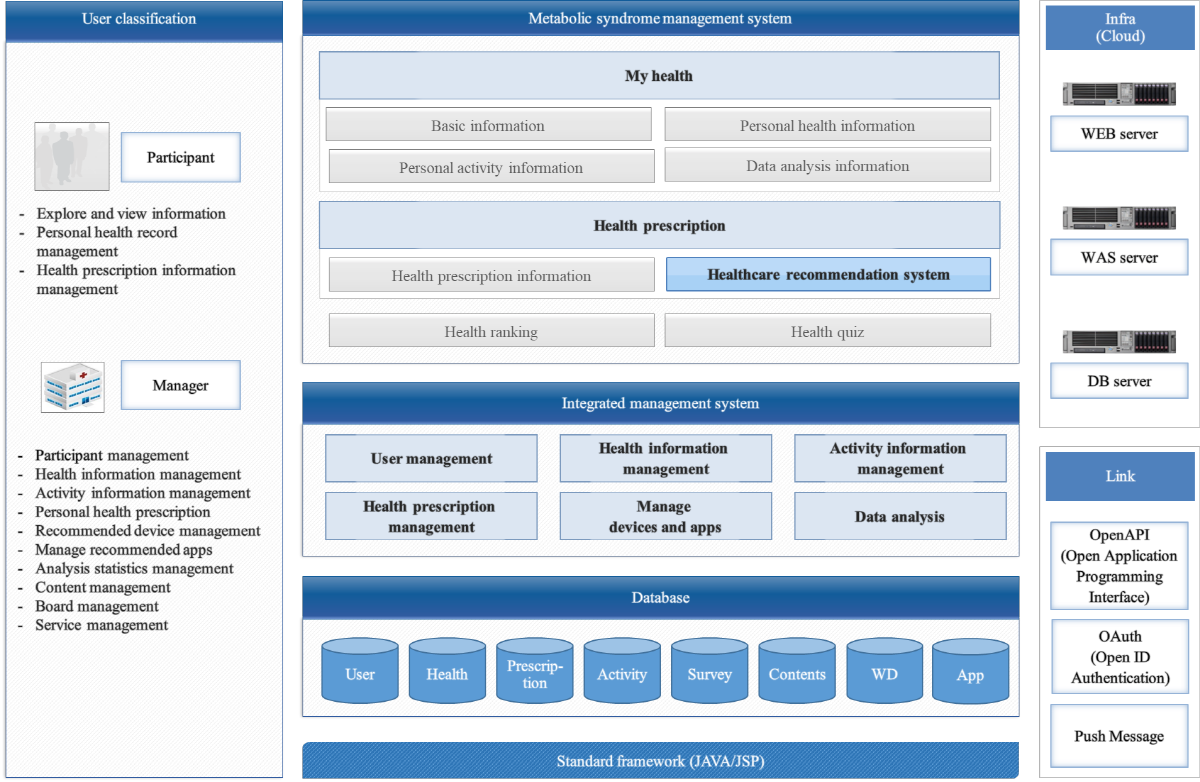
E-Motivate4Change system architecture.

**Figure 4:**
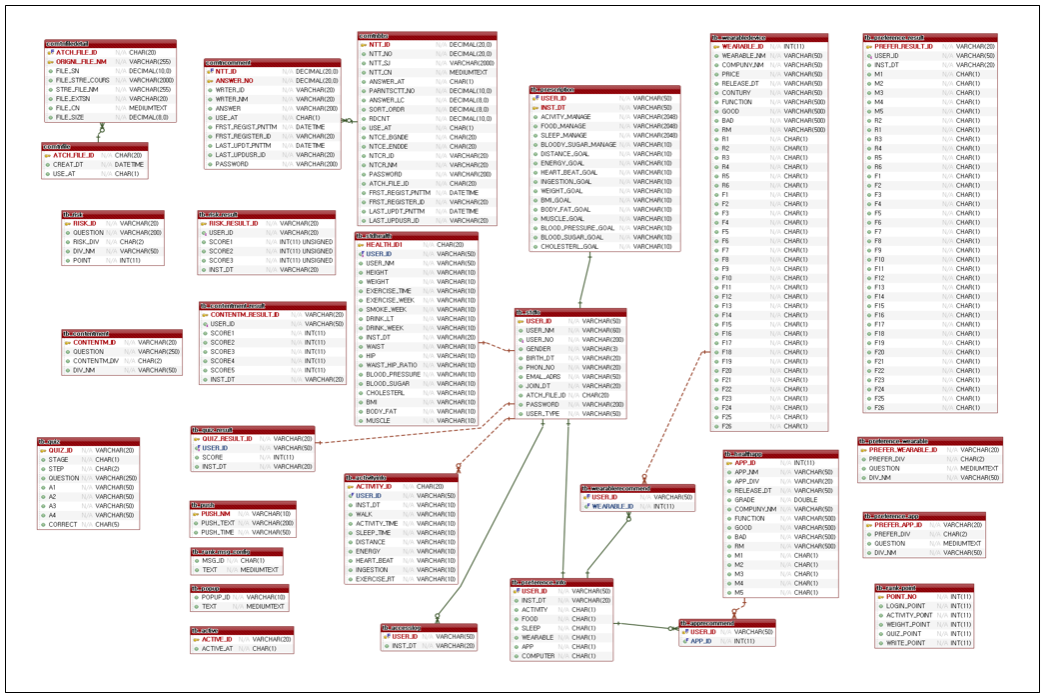
Entity-relation diagram of e-Motivate4Change system.

When participants entered their personal information, the e-Motivate4Change program generated and delivered tailored information and feedback. Such an interaction aimed to increase users’ interest and self-efficacy. The e-Motivate4Change program framework is shown in Figure 3. The entity-relation of the e-Motivate4Change system is shown in Figure 4.

To develop an effective intervention program for young adults with a risk of MetS, we surveyed and analyzed users’ demands, which could be used as foundational data for the development of further prevention and intervention programs that use mobile health. According to the needs analysis of professional program developers (N=40), nearly half had previously used health apps. The need for tailored prescription for individual users was rated the highest. Furthermore, 13.9% of the sample had used smart health bands. The most requested functions included a record of calorie consumption (88.9%), followed by a pedometer function, analysis of sleeping patterns, and a waterproof device [40].

We required a mobile app for data collection, a back-end server for storage, a server for real-time data analysis, a prediction server, and a message push server for a long-run comprehensive platform. To make exercise an entertaining factor, gamification was necessary. Therefore, we motivated users through the most foundational flow of gamification: mission, point, rewards [41]. After unifying all necessary factors, we developed a customized program for individual users [42].

**Figure 5:**
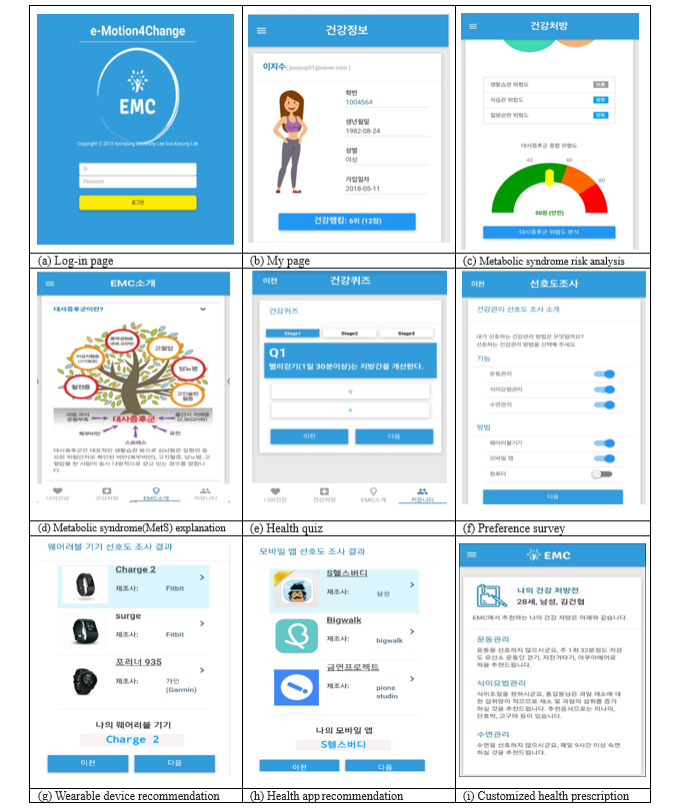
Screenshots of the e-Motivate4Change program.

The e-Motivate4Change program was developed based on three modules (Figure 5). The first is a health behavior prediction and planning module that notified users of their current risk of MetS when they input their current health status, life habits, and hobbies. It also provided customized health activity information (physical activity, diet, education, etc). In addition, it recommends a customized wearable device for users so that they can select one that they prefer. The second is an education module, which aimed to change users’ health behavior based on the I-Change 2.0 model [43]. The education module included the definition of MetS and its causes, diagnosis criteria, incidence rate, further symptoms, prevention, and intervention. Such contents were also customized to maintain users’ focus. The third module is an interactive communication module. Unlike previous one-sided feedback, the e-Motivate4Change program was designed to provide interactive feedback so that its users can share information and achieve the same goals together.

On the first page, participants could see a brief description about MetS, as well as a description of the program, types of MetS, and its risk factors. We also provided participants with links to websites (including YouTube) that provided information about effective dance moves for preventing MetS. Users could self-diagnosis their health status at the login page and check their risk of MetS as well as their body image. Risks of MetS were categorized into diet, disease, and lifestyle factors. Once the users answered a quick survey, the program provided scores on diet, disease, and lifestyle, indicating users’ risk of MetS as “fair,” “warning,” or “danger.”

**Figure 6:**
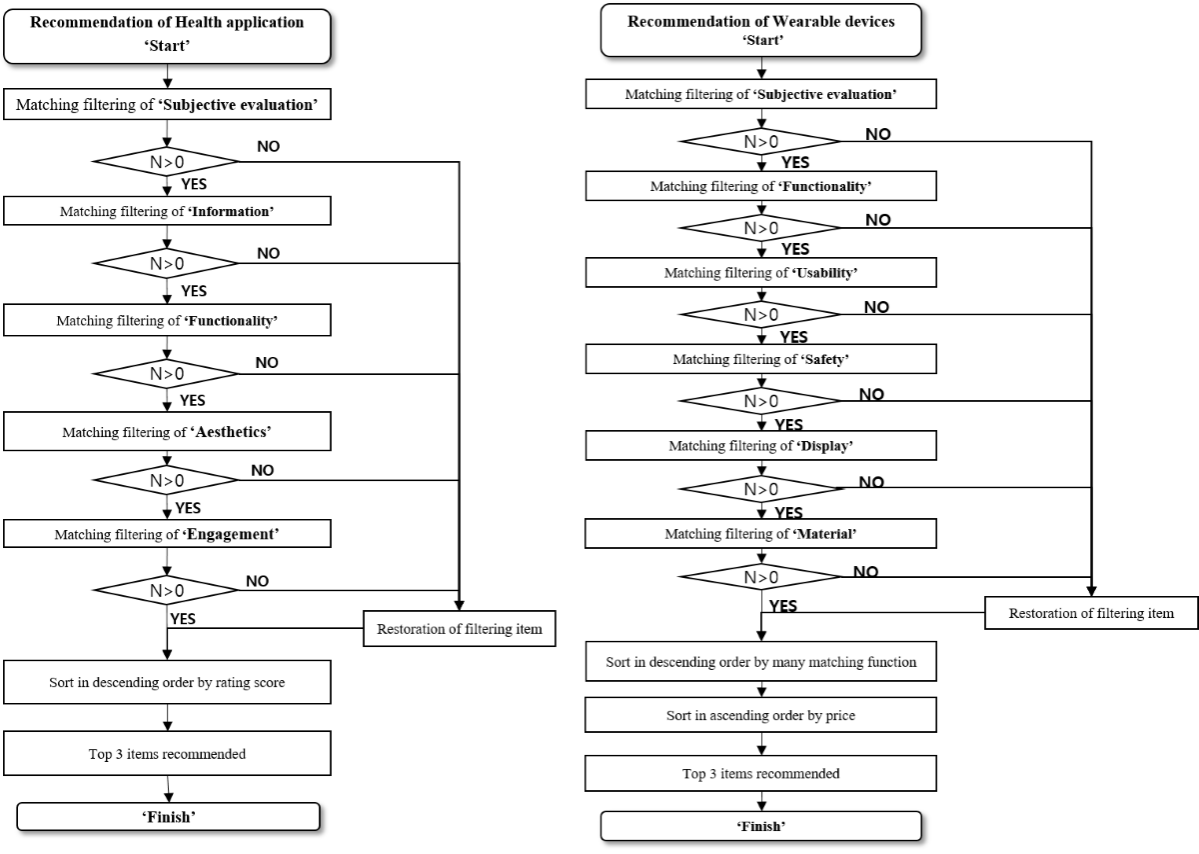
Recommended algorithm of e-Motivate4Change system.

Users were also asked to enter their basic health status, which provided an appropriate avatar with the same body image as the user (based on height and weight). There were five types of male and female avatars based on users’ risk of MetS, and the avatar was designed to change as users’ health status changed. Moreover, users could choose their preferred health management method (app, wearable, diet, or physical activity). The program reflected the needs of the users and recommended customized apps and wearable devices. The recommended algorithm is shown in Figure 6.

Concerning users’ health management, we sought the advice of two professional clinical nurses who worked at the national health care company, “Meta Health.” We regularly examined users’ health information such as weight, obesity rate, body fat, and fasting cholesterol to provide individualized health prescriptions, which included detailed descriptions regarding physical activity, diet, and sleep management.

The community category consisted of a social networking service, questions, and games and quizzes for entertaining factors. Health rankings and a free bulletin board represented the community function. First, users could view their real-time health ranking information, which was determined by points (“mileage”) gained from active participation in the program, such as writing on the bulletin or participating in a quiz. Users received mileage for getting the correct answer on a health quiz as well as writing and replying on the bulletin. To encourage participation and motivate the users, the users could check their real-time mileage rankings as well as their rewards. The program also included a “real-life alarm service” so that users could look at their avatars as their risk of MetS changed. Group messaging through a social network was also provided so that the participants could actively engage in information sharing about their health status. Lastly, we included a user preference survey so that we could receive feedback on their experience with the program at the end of the study.

### Statistical Analyses

All data collected through the study were analyzed using SPSS Statistics (Version 23.0; IBM Corp). To compare the general characteristics between groups, we used numbers, percentages, means, and standard deviations. We conducted chi-squared tests and Fisher exact tests to analyze the differences between groups, and we used *t* tests to determine if there were original differences in the dependent variable before the study. Subsequently, we used independent *t* tests to identify the effects of the program on participants’ healthy lifestyle promotion, self-efficacy, and QOL. A repeated-measures analysis of variance was conducted for participants’ physiological measurements (ie, weight, BMI, cholesterol, fasting blood sugar, and blood pressure level).

### Ethical Considerations

This study was approved by the heads of two universities in D city and by the Institutional Review Board of Keimyung University (number 40525-201704-HR-020-02). Participants were informed about the research purpose and methods, as well as their rights, including the right to cease participation at any point without penalty. All questionnaires were completed anonymously, and participants were told that the results would not be used for anything other than research purposes. Participation was voluntary, and all respondents provided written informed consent.

## Results

### Participants’ General Characteristics and Homogeneity Test

In total, 59 students (57 females) participated in the study (30 in the experimental group and 29 in the control group). Everyone agreed that lifestyle habits matter for the prevention of MetS. Further, 54.2% (26/48) answered that MetS was “a disease related to obesity and high cholesterol,” 75% (36/48) answered that MetS causes “adult disease,” 25% (12/48) answered that MetS results in “decreased physical strength and abnormal symptoms,” and 52.1% (25/48) answered that exercise matters the most for preventing MetS, followed by a better diet (20/48; 41.7%). No significant differences were found in the homogeneity test conducted between the experimental and control groups regarding general characteristics; thus, the two groups were homogeneous.

### Effect of the e-Motivate4Change Program on Psychosocial Indicators

We compared healthy lifestyle promotion, self-efficacy, and QOL between the two groups (Table 1). After the program, there were significant differences in healthy lifestyle promotion and self-efficacy, but not QOL. We also used Cronbach α to measure the reliability of the questionnaire.

**Table 1:** Comparison of psychosocial indicators between the experimental and control groups.

Variable and group		Prestudy, mean (SD)	Poststudy, mean (SD)	*t* value	*P* value
**Healthy lifestyle promotion**					
	Experimental (n=30)	173.13 (21.89)	191.57 (19.19)	3.86	<.001
	Control (n=29)	161.66 (26.12)	165.48 (24.76)	N/A^a^	N/A
**Quality of life**					
	Experimental (n=30)	88.73 (9.79)	88.50 (11.23)	.08	.93
	Control (n=29)	82.66 (9.28)	83.55 (9.80)	N/A	N/A
**Self-efficacy**					
	Experimental (n=30)	977.97 (185.87)	1140.33 (99.74)	6.00	<.001
	Control (n=29)	940.34 (178.11)	957.24 (134.66)	N/A	N/A

^a^N/A: not applicable.

### Effect of the e-Motivate4Change Program on Physiological Indicators

Table 2 shows participants’ change in BMI, cholesterol, blood sugar, and blood pressure based on different measurement periods. First, there were significant differences in BMI between groups and times; however, the interaction between group and time was nonsignificant. Second, there were significant differences in cholesterol scores per time, group, and the group × time interaction. Lastly, there were no significant differences concerning fasting blood sugar, systolic blood pressure, or diastolic blood pressure between groups.

**Table 2:** Comparison of physical and physiological indicators between the experimental and control groups.

Variable and group		Baseline, mean (SD)	Week 4, mean (SD)	Week 8, mean (SD)	Week 12, mean (SD)	*F* value (*P* value)		
						Group	Time	Group × time
**Cholesterol**								
	Experimental (n=30)	219.47 (66.09)	193.67 (53.84)	183.23 (52.62)	166.30 (43.15)	4.32 (.42)	9.73 (.001)	6.15 (.01)
	Control (n=29)	221.48 (61.01)	224.76 (63.41)	212.03 (60.30)	209.86 (58.98)	N/A^a^	N/A	N/A
**Body mass index**								
	Experimental (n=30)	23.34 (3.57)	23.24 (3.62)	22.67 (3.55)	22.10 (3.47)	1.01 (<.001)	4.71 (.03)	2.66 (.10)
	Control (n=29)	22.07 (3.28)	21.96 (3.14)	22.36 (3.62)	21.73 (3.34)	N/A	N/A	N/A
**Fasting blood sugar**								
	Experimental (n=30)	87.97 (12.35)	81.50 (9.13)	80.73 (8.53)	84.47 (7.87)	1.13 (.29)	1.99 (.11)	1.07 (.34)
	Control (n=29)	86.14 (6.36)	83.86 (22.65)	85.97 (9.80)	86.14 (6.36)	N/A	N/A	N/A
**Blood pressure (systolic)**								
	Experimental (n=30)	110.80 (10.17)	111.23 (11.27)	109.37 (11.737)	110.80 (10.17)	.55 (.46)	.49 (.66)	.13 (.94)
	Control (n=29)	109.62 (10.59)	109.34 (8.47)	108.52 (6.99)	108.52 (9.95)	N/A	N/A	N/A
**Blood pressure (diastolic)**								
	Experimental (n=30)	73.07 (9.79)	73.47 (10.37)	73.47 (10.37)	70.63 (7.60)	1.26 (.26)	1.06 (.37)	1.79 (.16)
	Control (n=29)	70.10 (8.40)	69.59 (6.81)	71.90 (6.82)	71.41 (6.06)	N/A	N/A	N/A

^a^N/A: not applicable.

## Discussion

### Principal Findings

To the best of our knowledge, this study was the first of its kind in Korea. This study was conducted to investigate the effectiveness of the e-Motivate4Change program using mobile apps and wearable devices developed to prevent and manage MetS in young adults. In total, 59 people from 2 universities in Korea participated. (experimental group n=30; control group n=29). The experimental group received a 12-week e-Motivate4Change program intervention, and the control group received MetS training and brochures without the e-Motivate4Change intervention. After the intervention, the experimental group scored significantly higher for health-related lifestyle (*t*=3.86; *P*<.001) and self-efficacy (*t*=6.00; *P*<.001) than the control group. With regard to BMI, there was a significant effect by group (*F*=1.01; *P*<.001) and group × time interaction (*F*=4.71; *P*=.03). Regarding cholesterol, significant main effects for group (*F*=4.32; *P*=.04) and time (*F*=9.73; *P*<.001) were confirmed.

As the boundaries of traditional industry change owing to the fourth industrial revolution, scholars are anticipating an era of individually tailored services [44]. Such a trend also applies to the national medical policy. The paradigm of worldwide medical policy is gradually shifting from treatment to prevention. Concurrently, considering the rising incidence rate of MetS, the need for appropriate prevention is necessary, especially among young adults who benefit most from lifestyle changes [14,17,45]. Thus, in this study, we developed a MetS prevention program that used mobile apps, wearable devices, and the advice of professional nurses and developers. We then tested its effectiveness on voluntary participants for 12 weeks.

Among both groups, most participants answered that lifestyle was a key preventive factor of MetS, and most defined MetS as a disease related to obesity and cholesterol. Many answered that there was an abnormal physical change when one fails to prevent MetS, and that exercise and diet improvement matter the most. Such answers are consistent with the results of Cornier and colleagues [16] and Clark and colleagues [21].

Participants were asked to record their daily activities in the e-Motivate4Change program for 12 weeks, and the program provided a visual change in the form of their avatars to display their goals and achievements. When we analyzed the risk factors of MetS, there was a significant difference in the mean change in BMI and cholesterol level between the two groups. There was a significant decrease in mean BMI and cholesterol for the experimental group. Such results also showed that, despite their young age, participants had a relatively high BMI and cholesterol level and were at a high risk for MetS [34].

For the experimental group, continued participation in the e-Motivate4Change program decreased their cholesterol and BMI significantly. This result aligns with Aktas and colleagues’ [46] study in Turkey, which tracked the lifestyles of participants for 12 weeks. These findings show that people with a chronic disease (including MetS) could lower their blood sugar and cholesterol by continuously improving their lifestyle. However, while fasting blood sugar and blood pressure levels decreased marginally for the experimental group, there was no significant difference between the two groups. One possible explanation is that all participants had average levels at baseline.

Concerning the psychosocial aspects of this study, we must note that the experimental group’s self-efficacy and healthy lifestyle promotion scores increased significantly. This result aligns with Khalili and colleagues [22] and Shekari and colleagues [47], who showed that a health education program based on social recognition theory increased young adults’ self-efficacy. However, when we look at QOL, the score of the experimental group slightly decreased, and there was no significant difference between the two groups. One potential explanation could be the short duration of the study. The study lasted for 12 weeks, which may have been too short to impact participants’ QOL. Therefore, further studies should implement a longer intervention to determine if it could increase young adults’ health-related QOL.

Moreover, it is critical to increase young adults’ self-efficacy and promote health-related lifestyles. Their needs and preferences should be properly understood so that more customized management programs can be developed. The e-Motivate4Change program is a customized interactive program that provides individualized information (ie, an avatar that reflects personal body shape and recommends health apps and wearable devices) and feedback based on users’ input. The program also used big data technology with gamification functions to entertain users, ultimately promoting a healthy lifestyle and educating users about MetS.

Generally, MetS can be prevented through changing diet habits and lifestyle. However, it can be challenging to maintain regular physical activities and good diet habits at an individual level. Thus, by recommending appropriate mobile apps and wearable devices and encouraging their use, this study made it possible for the users to engage in sustained health management. The findings of this study align with those from previous studies that revealed the significance of health trackers and smart apps that are free of time and space limitations for a sustained increase in physical activities [30,48]. Previous recommendation systems were rather one-sided and manager-centered, which made it difficult for users to stay entertained. Consequently, most users stopped using the service.

To find a solution, we developed a needs-based customized recommendation system. Some of the most important features of this program can be summarized as follows. First, the program provides individualized health management prescriptions for the prevention of MetS. Based on the medical paradigm shift from treatment to prevention and management owing to an increasing risk of chronic disease, effective prevention is largely changing the clinical health sectors, as is the fourth industrial revolution [45]. Thus, the combination of data and artificial intelligence will enable individualized medical service. When supported with digital technology, the prevention and management of chronic disease will become much more effective and efficient [45,49]. Furthermore, it is critical for health managers to learn new competencies and skills in rapidly changing medical environments [49]. Thus, health managers for the e-Motivate4Change program analyzed the real-time data of participants and provided customized consulting and health prescriptions, which brought about positive changes in health-related lifestyle and self-care. An efficient system will increase the usability of medical services, decrease the symptoms of chronic disease, and increase cost efficiency, while also improving patients’ medical experiences.

Clinical nurses play a critical role in enhancing health; therefore, more effort is needed to expand the role of nurses and support their competency [50]. Clinical nurses will pioneer a new field of expertise by using big data technology and artificial intelligence. Thus, it is necessary to combine the humane and technological aspects of nurse’s expertise [45,49,51].

Second, previously one-sided services may not include gamification and entertainment factors. Such improvement provides individualized services for the users, maintaining their high level of interest and their motivation, encouraging them to keep using the program. For sustained management of chronic diseases, including MetS, more user-centered content is necessary.

### Limitations

The personalized service recommendation system proposed in this study provides appropriate dietary and physical activity recommendations to improve youth health to prevent MetS. The ranking system included in the program provides entertainment and motivation to encourage continued use of the program. Moreover, this study can be regarded as different from previous studies as the participants were aged <30 years, whereas the typical age range of MetS study participants is 40 to 60 years. However, this study had limitations. We used a portable body fat measurement device that may not be able to provide accurate and detailed data. At the same time, it was not possible to monitor the type, intensity, or length of physical activity. Subsequent studies should aim to achieve this. In addition, future studies could aim to recruit a participant group with a wider age range than this study. For example, future health intervention programs could include young working women and working mothers.

### Conclusions

Despite its limitations, this study is meaningful in that we tried to increase accessibility and usability through smartphone apps while also bringing about changes in the perceptions and health behaviors of young adults by providing individualized recommendations from professional clinical nurses [47]. Moreover, unlike previous group-based education, this program encouraged users’ voluntary participation in creating a healthy lifestyle, which allows for better long-term management and operation of the program.

In conclusion, the e-Motivate4Change program was associated with increased physical activity, decreased BMI, lower cholesterol, and increased self-efficacy among experimental group participants, thus effectively promoting a health-related lifestyle. Moreover, by providing programs to prevent and manage MetS, the program informed further studies. Therefore, this study provides foundational data to design further preventive strategies for relevant chronic disorders (eg, diabetes, high blood pressure, hyperlipidemia, cardiovascular disease).

## Abbreviations

MetS: metabolic syndrome
QOL: quality of life
WHO: World Health Organization
